# A case report of inguinal hernia combined with sparganum infestation

**DOI:** 10.1093/jscr/rjaf784

**Published:** 2025-10-08

**Authors:** Jingle Huang, Shen Li, Lixiu Huang, Huiying Li, Zhendong Qiu, Zhixing Lu, Sheng Xu, Chao Liu, Jinlei Wei

**Affiliations:** Surgery for Gastrointestinal Hernia and Fistula Nanning, People's Hospital of Guangxi Zhuang Autonomous Region, No. 6, Taoyuan Road, Qingxiu District, Guangxi Zhuang Autonomous Region, 530000, China; Surgery for Gastrointestinal Hernia and Fistula Nanning, People's Hospital of Guangxi Zhuang Autonomous Region, No. 6, Taoyuan Road, Qingxiu District, Guangxi Zhuang Autonomous Region, 530000, China; Medical Laboratory Department Nanning, The First Affiliated Hospital of Guangxi Medical University, No. 6, Shuangyong Road, Qingxiu District, Guangxi Zhuang Autonomous Region, 530000, China; Education Department Nanning, The First Affiliated Hospital of Guangxi Medical University, No. 6, Shuangyong Road, Qingxiu District, Guangxi Zhuang Autonomous Region, 530000, China; Surgery for Gastrointestinal Hernia and Fistula Nanning, People's Hospital of Guangxi Zhuang Autonomous Region, No. 6, Taoyuan Road, Qingxiu District, Guangxi Zhuang Autonomous Region, 530000, China; Surgery for Gastrointestinal Hernia and Fistula Nanning, People's Hospital of Guangxi Zhuang Autonomous Region, No. 6, Taoyuan Road, Qingxiu District, Guangxi Zhuang Autonomous Region, 530000, China; Surgery for Gastrointestinal Hernia and Fistula Nanning, People's Hospital of Guangxi Zhuang Autonomous Region, No. 6, Taoyuan Road, Qingxiu District, Guangxi Zhuang Autonomous Region, 530000, China; Surgery for Gastrointestinal Hernia and Fistula Nanning, People's Hospital of Guangxi Zhuang Autonomous Region, No. 6, Taoyuan Road, Qingxiu District, Guangxi Zhuang Autonomous Region, 530000, China; Colorectal and Anorectal Surgery Nanning, People's Hospital of Guangxi Zhuang Autonomous Region, No. 6, Taoyuan Road, Qingxiu District, Guangxi Zhuang Autonomous Region, 530000, China

**Keywords:** Spirometra mansoni, sparganum；parasite, inguinal hernia

## Abstract

Inguinal hernia is a common benign disease in general surgery. It is rare to find Spirometra mansoni sparganum in the preperitoneal space during surgery. Most patients acquire the infection through consumption of raw frog or snake meat, application of snake skin on the skin or wounds. Patients may experience subcutaneous itching, a sensation of moving parasites, and even symptoms such as redness, swelling, heat, and pain. Most patients can be cured through comprehensive treatment combining oral medication and surgical intervention. Strengthening public education and promoting behavioral changes, including healthier dietary and lifestyle habits, are effective measures for preventing this condition.

## Introduction

Sparganum is the larva of Spirometra mansoni. The worm body appears as a white, elongated ribbon and possesses strong contractile capabilities. The eggs are excreted into water along with the host’s feces and, under suitable conditions, hatch into coracidia. After being ingested by the first intermediate host, Cyclops, the coracidia develop into procercoids within its body. When the Cyclops containing procercoids is consumed by the second intermediate hosts such as frogs and snakes, the procercoids develop into sparganum. When the definitive hosts such as cats and dogs ingest the second intermediate hosts containing sparganum, the sparganum develop into adult worms in their intestines. If humans accidentally consume food containing sparganum or come into contact with contaminated items, they may also be infected with sparganum. Sparganum can be distributed in multiple parts of the human body, causing sparganosis and posing a threat to human health [1]. Sparganum infestation in the preperitoneal space concurrent with inguinal hernia is rare. We report one such case treated in the Department of General Surgery at the People’s Hospital of Guangxi Zhuang Autonomous Region, and present the findings below.

## Presentation of case

**Figure 1:**
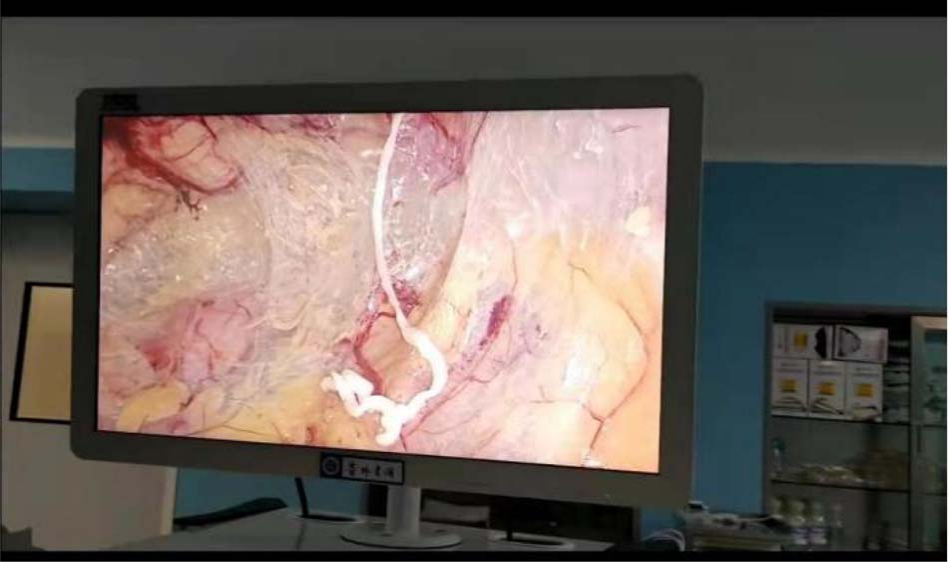
Preperitoneal space white, elongated, motile parasitic worm.

**Figure 2:**
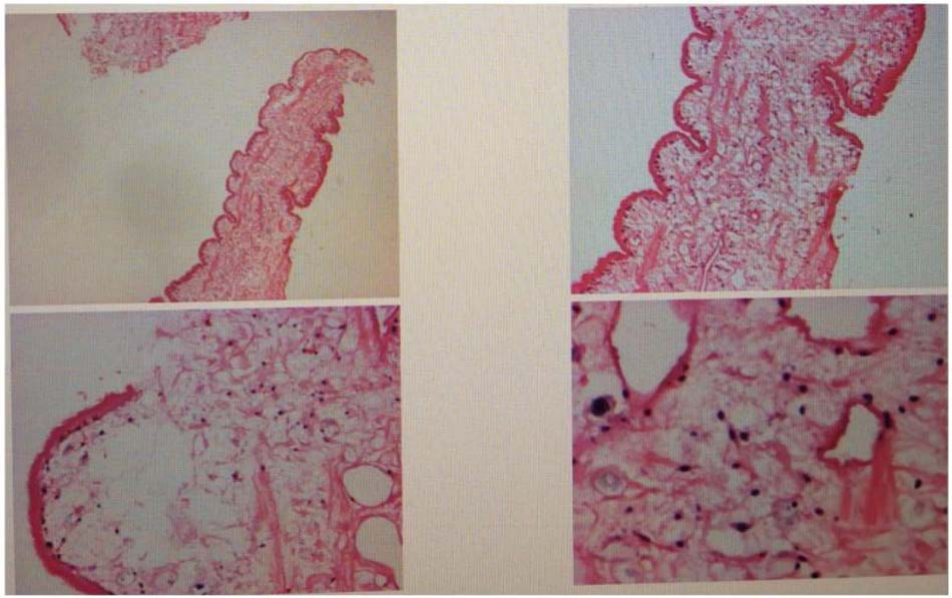
Postoperative pathology indicates that the submitted specimen is a parasitic organism.

**Figure 3:**
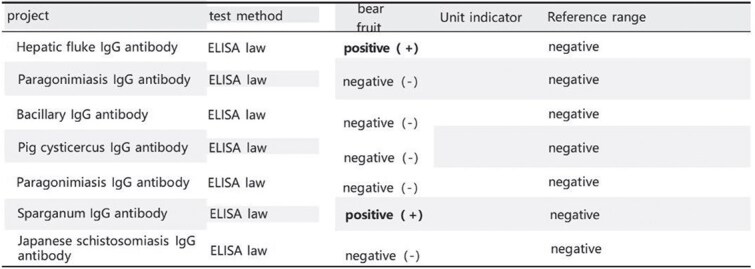
Serum immunological examination: Sparganum ELISA (+).

The patient was a 48-year-old male admitted due to a ‘right inguinal mass for 40 years with worsening and abdominal pain for 1 month.’ He had a history of consuming raw freshwater shrimp, crabs, and snake meat. Upon admission, physical examination revealed stable vital signs. Local examination showed a soft, ⁓6 × 5 cm right inguinal mass without significant tenderness, which disappeared when lying down.Laboratory tests: Complete blood count showed white blood cell count of 5.96 × 10^9^/L; granulocyte percentage of 43.5%; hemoglobin level of 142 g/L; eosinophil percentage of 2.0%; platelet count of 317 × 10^9^/L. Routine stool examination did not detect parasite eggs. Color Doppler ultrasound of bilateral inguinal regions and scrotum revealed an inhomogeneous echoic mass in the right inguinal area, consistent with inguinal hernia measuring ⁓6 × 5 cm. All other examinations were unremarkable. Preoperative preparations were completed, and laparoscopic right inguinal hernia repair was performed. During surgery, a white, elongated, motile parasite (⁓10 cm in length) was found within the preperitoneal space in the Hesselbach triangle (Fig. 1). This parasite is entirely white in color, having a flat and slender shape. There are no visible legs, and it is impossible to distinguish the head from the tail. Its movement is very slow (Video). Postoperative pathology reported two parasite specimens from the right inguinal region, measuring 1.6 cm and 7.0 cm in length respectively, with a width of 0.2 cm each (Fig. 2). Serum immunological examination (Fig. 3): Sparganum enzyme-linked immunosorbent assay (ELISA) (+). Follow-up via telephone after discharge indicated good surgical recovery; no pain or swelling in the right inguinal region, and no protruding mass.

## Discussion

Finding spargana in the preperitoneal space during inguinal hernia surgery is extremely rare. The patient had a history of consuming raw freshwater shrimp, crabs, and snake meat, which is likely related to his sparganosis [1]. Spargana are the plerocercoid larvae of Spirometra mansoni, whose adult forms reside in the intestines of cats and dogs, and occasionally parasitize the human intestine.Spargana are elongated, ribbon-like, white parasites ranging in length from 0.3 cm to 105 cm [2]. They commonly parasitize the muscles of frogs and the subcutaneous tissue of snakes [3]. When infected frogs or snakes are ingested by paratenic hosts such as birds, pigs, or humans, the spargana do not develop into adult worms within the digestive tract [4, 5]. Instead, they penetrate the intestinal wall and migrate to the abdominal cavity, muscles, or subcutaneous tissues, where they continue to survive but cannot mature into adults [6]. Sparganosis is a severe parasitic infection caused by the larvae of Spirometra mansoni, also called ‘sparganum.’ In human hosts, the Spirometra mansoni larva commonly targets the subcutaneous tissue or muscle. Sometimes it can also migrate into the brain [7]. Additionally, serum immunological testing for sparganosis revealed a positive result using a rapid enzyme-linked immunosorbent assay specific for Spirometra mansoni, aiding in diagnosis. The most important and reliable diagnostic method is identifying the parasite species through morphological and molecular analysis of the recovered specimen. Based on the patient’s history of consuming raw freshwater shrimp, crabs, and snake meat, along with intraoperative observation of a parasitic organism in the preperitoneal space, the diagnosis of sparganosis was confirmed. For similar cases suspected of sparganosis infection, detailed epidemiological history should be obtained, and early diagnosis and treatment initiated. Public education is also essential—avoid applying raw frog or snake meat to skin or wounds, and refrain from consuming raw amphibian or reptilian meats as effective preventive measures against this disease.

## Supplementary Material

Video_of_Spirometra_mansoni_sparganum_rjaf784
